# Cervical Osteophyte as a Rare Etiology of Spontaneous Intracranial Hypotension With Bilateral Subdural Hematomas: A Case Report and Literature Review

**DOI:** 10.7759/cureus.106674

**Published:** 2026-04-08

**Authors:** Fatima El Kartit, Daniel Yamba Yamba, Jihad Mortada, Robin Srour

**Affiliations:** 1 Neurosurgery, Mohammed VI University Hospital Center, Oujda, MAR; 2 Neurosurgery, Hôpitaux Civils de Colmar, Colmar, FRA; 3 Neurosurgery, Hassan II University Hospital of Fez, Fez, MAR

**Keywords:** bone spur, dural breach, orthostatic headaches, spontaneous intracranial hypotension, young adult

## Abstract

Spontaneous intracranial hypotension (SIH) is a clinical syndrome typically resulting from occult cerebrospinal fluid (CSF) leaks, though a cervical bone spur as the primary etiology is an exceptionally rare occurrence. This report describes a 41-year-old male patient who presented with a two-month history of persistent headaches, dizziness, and an acute confusional state. Initial imaging revealed bilateral subdural hematomas, while subsequent magnetic resonance imaging (MRI) of the brain and spine identified diffuse meningeal enhancement and a type 1 meningeal breach at the C7-T1 level caused by a focal osteophyte. The patient underwent a successful two-stage surgical intervention involving an anterior cervical discectomy and fusion (ACDF) to repair the dural defect, followed by the evacuation of the hematomas. Post-operative recovery was uneventful, with a six-month follow-up confirming the complete resolution of intracranial abnormalities and a full return to functional baseline. This case emphasizes that SIH should remain a diagnostic consideration in patients with persistent headaches and underscores the importance of identifying mechanical spinal triggers, such as osteophytes, to ensure definitive surgical repair and optimal neurological outcomes.

## Introduction

Spontaneous intracranial hypotension (SIH) is a recognized but often underdiagnosed condition. It is usually characterized by orthostatic headaches linked to low cerebrospinal fluid (CSF) pressure and no history of previous dural trauma or invasive treatment on the spine [1]. The headaches typically occur within 15 minutes of standing and resolve within 30 minutes of lying down. Less specific symptoms include hearing impairment, cranial nerve damage, nausea, and vomiting [2]. A range of neuroimaging modalities, including magnetic resonance imaging (MRI) of the brain and spine, computed tomography (CT) of the head and spine, as well as myelography, may be employed to support the diagnosis, determine the underlying etiology, and accurately localize the site of dural disruption. These investigations are essential for guiding the development of an appropriate therapeutic strategy [3,4].

The treatment approach varies based on the underlying cause. For most patients, a conservative method with bed rest and hydration is preferred. When conservative treatment does not work, more advanced options, such as blood patches or surgical procedures to repair dural defects or CSF venous fistulas, may be considered, although these procedures are rarely performed [5].

CSF leakage is an uncommon consequence of degenerative spinal bone pathology [6]. A comprehensive review of the literature identified only three studies, collectively reporting five cases of SIH secondary to a cervical osteophytic lesion [7,8]. The rarity of such presentations frequently contributes to diagnostic challenges, with many patients initially receiving incorrect diagnoses and experiencing delays ranging from days to months before an accurate diagnosis is established. Furthermore, the limited number of reported cases of this uncommon etiology of SIH has resulted in a paucity of evidence regarding optimal strategies for early diagnosis and management [9,10].

In this context, we present a rare case of SIH caused by a cervical osteophyte in a healthy young male patient who posed significant diagnostic difficulty. This report aims to increase awareness of similar presentations and to highlight cervical degenerative spinal bone disease as an important potential cause of SIH, even within younger populations.

## Case presentation

A 41-year-old man with no significant past medical history or recent history of trauma presented to the emergency department with a two-month history of persistent headache accompanied by dizziness and an acute confusional state, characterized over the past four days by difficulty focusing on topics, sluggishness, and speech disturbances or difficulty recalling words.

Three weeks before his admission to the emergency department, he was seen by his doctor for headaches, neck pain, asthenia, and fever. A full blood test showed leukocytosis and an elevated C-reactive protein (CRP) level. Blood cultures were negative for infectious organisms. A brain CT scan showed no abnormalities. Due to a perturbed blood count, his doctor considered these headaches to be simple flu-like symptoms and treated them with analgesics and antibiotics, without improvement.

The patient was then examined by a neurologist one week later due to ongoing headaches, impaired attention, and a regressive episode of diplopia. The neurologist suspected autoimmune encephalitis, so a lumbar puncture was performed. The test results were normal, and the patient was awaiting a brain MRI.

**Figure 1:**
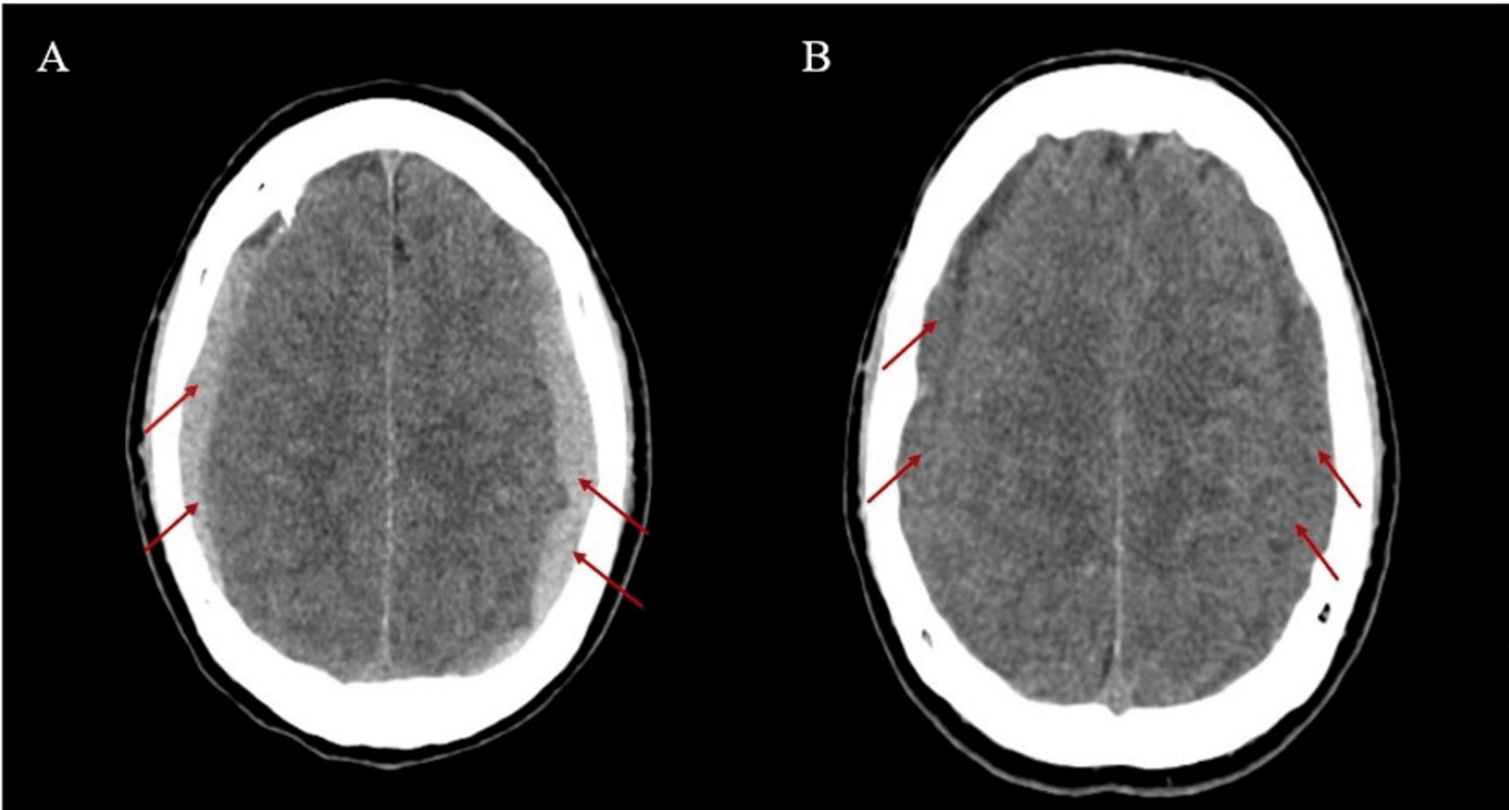
First and second brain CT scan The initial brain CT scan shows a bilateral subacute subdural hematoma (red arrows) (A). The second scan shows a bilateral chronic subdural hematoma (red arrows) (B). CT: computed tomography

On admission to the emergency department, the patient was confused and mildly drowsy, with a Glasgow Coma Scale (GCS) score of 13 with an acute confusional state and no focal neurological deficits on examination. Vital signs were stable, including a blood pressure of 120/70 mmHg, a heart rate of 70 beats per minute, and a capillary blood glucose level of 1.2 g/L. A non-contrast brain CT scan demonstrated bilateral subacute subdural hematomas measuring 9 mm in thickness on the right and 10 mm on the left (Figure 1). He was admitted to the neurosurgery department for clinical monitoring.

**Figure 2:**
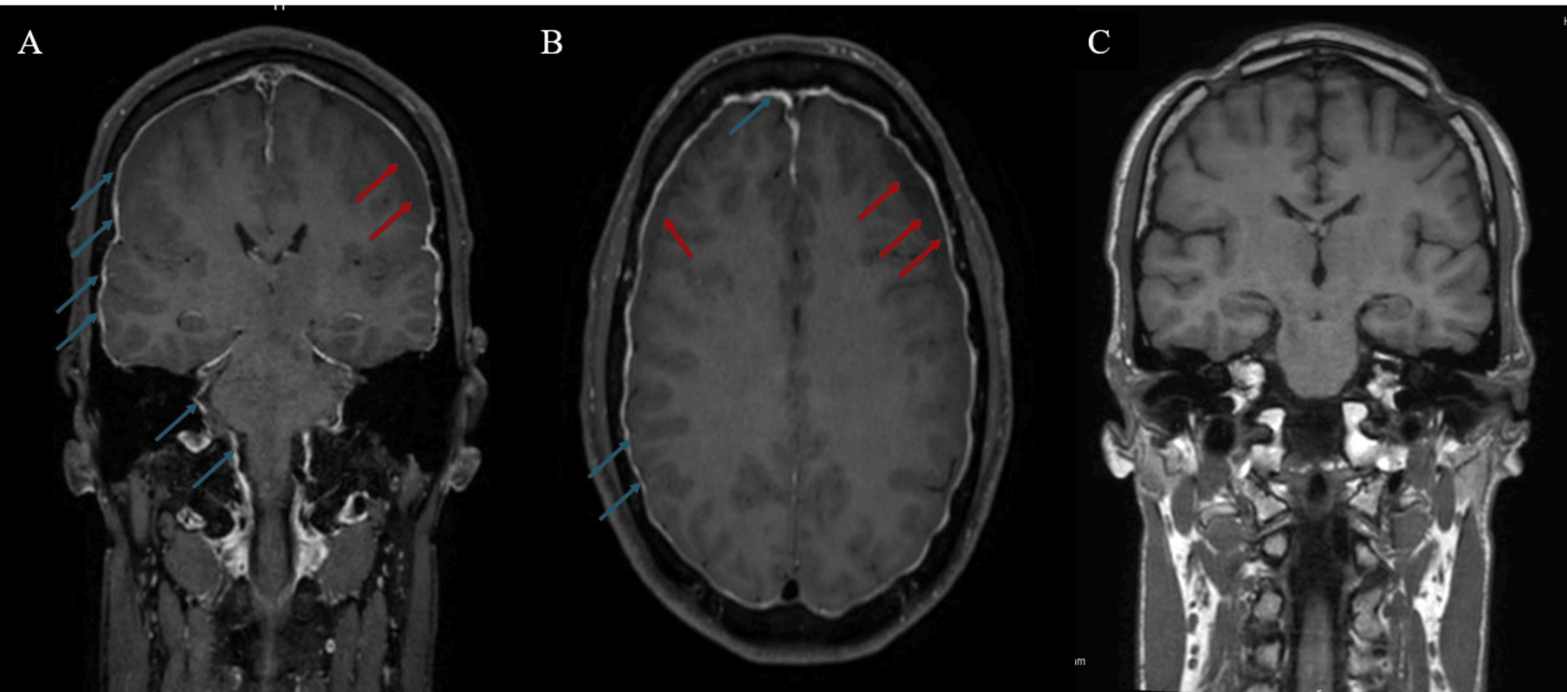
Pre-operative and six months post-operative brain MRI Spontaneous intracranial hypotension. (A) Coronal and (B) axial pre-operative T1-weighted gadolinium-enhanced MRI demonstrate diffuse pachymeningeal enhancement (thin blue arrows), brainstem sagging, and bilateral subdural collections (thin red arrows). (C) Post-operative T1-weighted gadolinium-enhanced coronal MRI shows a complete resolution of pachymeningeal enhancement and bilateral subdural collections. MRI: magnetic resonance imaging

During hospitalization, on day 4, his condition worsened, and the patient became increasingly drowsy, with acute neurological deterioration to a GCS score of 10. A brain MRI was then performed, showing diffuse meningeal enhancement with brainstem sagging apart from the bilateral subdural hematoma (Figure 2).

The diagnosis of SIH was made based on the results of the brain MRI. Subsequently, a series of imaging studies were performed on the same day to investigate the etiology of SIH.

**Figure 3:**
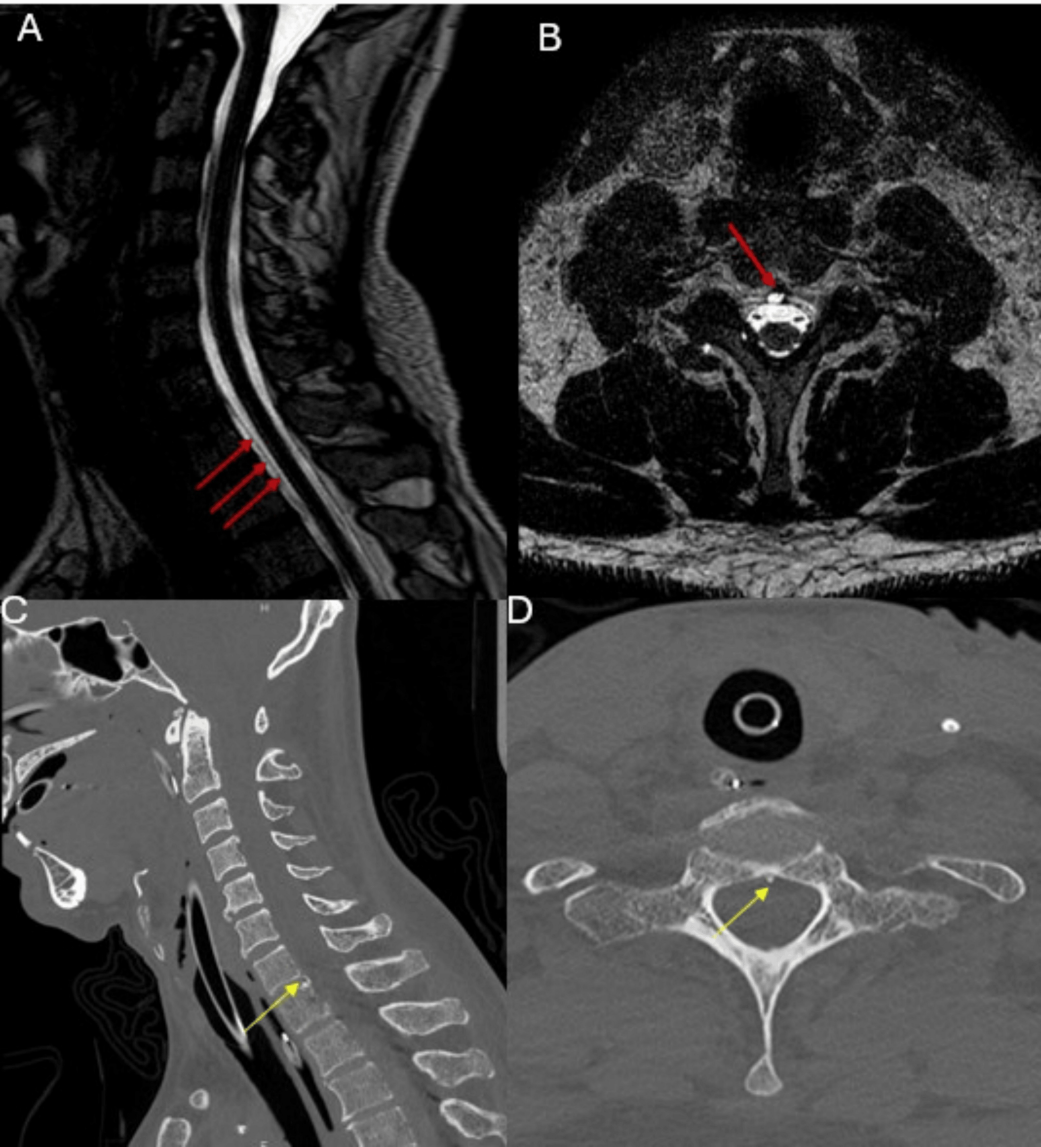
Cervical MRI and CT scan Spinal longitudinal extradural collections (red arrows) are demonstrated on sagittal T2-weighted fast spin-echo (A) and axial T2-weighted (B) MRI. Sagittal (C) and axial (D) cervical spinal CT images reveal a rostrally oriented osteophyte at the C7-T1 level (yellow arrows). MRI: magnetic resonance imaging; CT: computed tomography

First, a spinal MRI was performed, revealing a fluid collection primarily in the anterior upper cervicothoracic region, with a strong suspicion of a type 1 breach at C7 at this level, associated with a C7-T1 bone spur (Figure 3). Second, a cervical CT scan revealed a sharply pointed osteophyte oriented rostrally at the C7-T1 level (Figure 3).

**Figure 4:**
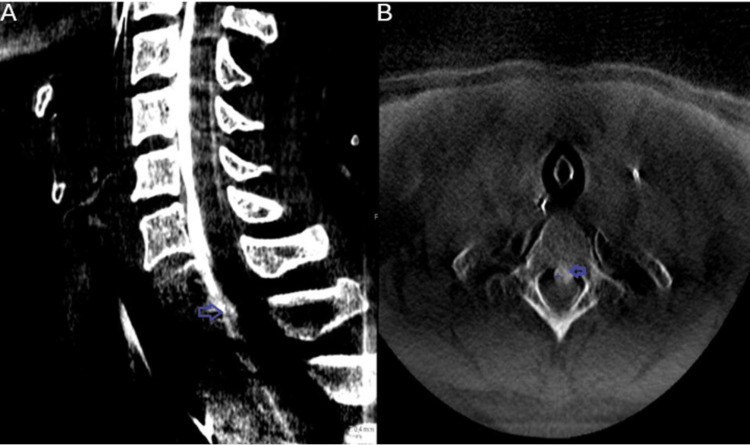
Cervical CT myelogram (A) Sagittal CT myelogram demonstrates a rostral osteophyte at the C7-T1 level with a gradient of myelographic contrast indicating a CSF leak (blue arrowheads). (B) Axial CT myelogram demonstrates the sharp, posteriorly directed osteophyte anterior to the spinal cord; the blue arrowheads show the epidural contrast collection ventral to the spinal cord. CT: computed tomography; CSF: cerebrospinal fluid

Finally, to confirm the location of the dural breach, CT myelography was performed, revealing a fluid collection at the cervicothoracic junction with progressive contrast leakage (Figure 4).

He underwent cervical surgery to repair the dural breach at the C7-T1 level through an anterior cervical discectomy and fusion (ACDF) at the same level. Upon resection of the posterior longitudinal ligament (PLL), a right-sided dural tear with an active CSF leak was identified along with the osteophyte. The defect was primarily managed using a TachoSil® fibrin sealant patch, which was further reinforced with Tisseel® fibrin glue to ensure a watertight closure. 

On post-operative day 1, the patient's symptoms improved along with his level of consciousness.

On post-operative day 10, the patient started experiencing transient consciousness disturbances, gradually becoming more drowsy with a GCS score of 12. The rest of the neurological exam was normal.

Within two hours of symptom, a repeat brain CT scan revealed bilateral chronic subdural hematomas, with the right hematoma increasing from 9 mm to 11 mm and the left from 10 mm to 14 mm (Figure 1).

A second surgery was performed on the same day to evacuate the bilateral subdural hematomas. The post-operative course was favorable, with a GCS score of 15 and no headaches. 

Three months after his hospitalization, the patient returned for a follow-up consultation. He no longer experienced headaches and had no previously reported symptoms. An MRI of the head and neck was performed, revealing the resolution of the subdural hematomas and disappearance of the meningeal enhancement (Figure 2).

## Discussion

Epidemiology

SIH was first described in 1938 by Georg Schaltenbrand as a syndrome in which CSF volume depletion can cause a variety of neurological symptoms [1]. This is a rare condition with an estimated incidence of five per 100000 per year, with a female predilection; the female-to-male ratio is 2:1, and it peaks in the fourth decade of life [2-5]. Although rare, it is increasingly recognized as a condition characterized by CSF leakage, most commonly in the thoracic spine [9-11]. Wang et al., in their latest study, proposed an updated SIH grading scale based on the patient's presenting symptoms, adding a fourth grade to the Arshad et al. score [12].

In our case, the patient is a Turkish man in his 40s with no history of trauma. He initially presented with a grade 1 condition, as his main complaint was persistent headaches, but it gradually worsened to a grade 4. The leakage was later identified at the cervicothoracic junction.

Pathophysiology and etiology

SIH, previously considered a pressure-based disorder, is now recognized as a volume-depletion syndrome caused by CSF leakage through the spinal arachnoid [13].

The pathophysiological mechanisms remain incompletely understood; three principal etiologies have been proposed: meningeal diverticula, CSF-venous fistulas, and dural breaches [14]. Among these, dural tears are most frequently associated with calcified disc herniations or sharp osteophytic spurs, which create linear longitudinal defects through the repetitive mechanical erosion of the dura mater. These lesions preferentially involve the thoracic and lower cervical spine, where disc calcification is more prevalent. The resulting CSF leakage is often brisk, leading to large and extensive epidural CSF collections [15]. In the present case, the predominant mechanism underlying SIH was a dural breach caused by a cervical osteophytic spur, resulting in extensive epidural CSF collections.

In accordance with the Monro-Kellie doctrine, the decrease in CSF volume is accompanied by compensatory venous engorgement and an increase in intracranial blood volume, further contributing to the painful stretching of neural and meningeal fibers [15]. Due to these compensatory mechanisms and traction, subdural hematomas are not uncommon in patients with SIH [1]. In our patient, this process manifested as bilateral subdural hematomas, which were successfully managed with a surgical approach.

SIH may arise in the absence of identifiable predisposing factors. Although current evidence remains limited, clinical evaluation may include assessment for underlying connective tissue disorders and joint hypermobility syndromes, as well as spinal pathology, such as osteophytes, intervertebral disc herniation, and discogenic microspurs, particularly when these are anatomically correlated with the site of CSF leakage [6].

Clinical aspects

The clinical manifestations of SIH are not fully understood yet, and patients with this condition can present a wide range of different complaints that are not always purely neurological. Throbbing orthostatic headaches associated with neck pain are the most frequently reported symptoms [9]. These headaches are usually localized to the occipital-nuchal or frontal areas and are thought to result from the sagging of brain structures downward, causing traction on the anchoring and pain-sensitive structures [5]. Jurcau et al. conducted a study over nine years and found that headaches were the initial symptom in 94% of patients [10]. Podkovik et al. performed a similar study over seven years and observed that headaches were present in 84% of patients [11]. These headaches typically occur shortly after rising and tend to lessen over time; they can be accompanied or overshadowed by other symptoms such as nausea, vomiting, auditory disturbances, visual disturbances, gait issues, speech abnormalities, or altered mental state that can range from confusion to coma. Some patients also report difficulty concentrating and fatigue [12].

Less specific symptoms make the diagnosis more difficult, reducing the likelihood of identifying a CSF leak. This was the case for our patient, whose headaches, although the main issue, were initially attributed to a flu-like illness or meningoencephalitis. His admission to the neurosurgery department was for evacuating the subdural hematomas rather than investigating a leak. In even rarer cases, the patient can present with cognitive symptoms (dementia), sleep palsy, cranial nerve palsy, or endocrine disorders such as hyperprolactinemia and galactorrhea, which can be explained by the gravitational forces applied to the pituitary stalk [11].

Diagnosis

As outlined above, the defining clinical feature of headache attributed to SIH is its orthostatic character. Nevertheless, this feature is not required by the diagnostic criteria of the International Classification of Headache Disorders, Third Edition (ICHD-3), as postural dependence may be attenuated or absent, particularly in chronic disease states or in the setting of low-flow CSF fistulae [16].

According to the ICHD-3, diagnosis requires fulfillment of the following criteria: (A) a headache meeting the criteria for headache attributed to low CSF pressure and criterion C; (B) absence of any procedure or trauma known to cause CSF leakage; (C) development of headache in temporal association with the occurrence of low CSF pressure or CSF leakage or headache leading to its detection; and (D) exclusion of alternative ICHD-3 diagnoses that better account for the clinical presentation [16].

Gadolinium-enhanced brain MRI is the neuroimaging modality of choice for confirming the clinical suspicion of SIH, with diagnostic findings reported in approximately 80% of cases [17]. The most common radiological feature is diffuse, homogeneous pachymeningeal enhancement [18]. However, accurate interpretation of these findings requires specialized expertise to avoid arbitrary conclusions, which has led to the development of standardized assessment tools, such as the Bern score [4].

Head CT is typically unremarkable, although reported abnormalities include engorgement of the venous sinuses, reduced ventricular size, subdural fluid collections, obliteration of the prepontine cistern, and imaging features consistent with pseudo-subarachnoid hemorrhage, such as hyperdensity along the tentorium and Sylvian fissures [17].

When SIH is suspected, spinal MRI should be performed concurrently with brain MRI [19]. Gadolinium-enhanced spinal MRI, including fat-suppressed T2-weighted sequences, may demonstrate pachymeningeal enhancement, the dilatation of nerve root sheaths, the engorgement of epidural venous sinuses, or the presence of meningeal diverticula [3]. In addition to indirect signs, spinal MRI may facilitate the localization of the CSF leak by identifying non-compressive epidural fluid collections, which are observed in approximately 60% of cases [19]. We performed a spinal MRI, including fat-suppressed T2-weighted sequences, showing a non-compressive epidural spinal longitudinal extradural CSF collection at the cervicothoracic level.

Lumbar puncture is not essential for the diagnosis of SIH and may, in fact, exacerbate the patient's clinical condition [4].

Treatment

There are several treatment options for SIH ranging from conservative treatment, epidural blood patch (EBP) and epidural fibrin glue, to surgical repair.

In 2023, Cheema et al. proposed consensus guidelines for the management of SIH [20].

Conservative Treatment

For patients with acute uncomplicated intracranial hypotension, we can suggest conservative measures hoping that the leak will resolve without the need for going into invasive techniques. Conservative management aims to both alleviate symptoms and prevent progression of the CSF leak. This approach includes bed rest to reduce headaches and facilitate spontaneous dural closure, adequate hydration, and avoidance of Valsalva maneuvers. For this reason, abdominal binders are often recommended. Notably, the efficacy of corticosteroids remains unproven, and their use may be associated with potential adverse effects. Caffeine and theophylline are adenosine antagonists; therefore, they can increase intracranial pressure and improve headaches. The evidence of efficacy for both is lacking, but in a double-blind, placebo-controlled trial, oral caffeine has been shown to improve post-dural puncture headaches [5].

Untargeted EBP: For CSF leaks that do not improve with conservative measures, several options exist, but the main treatment is large-volume, non-targeted lumbar EBP, performed at the junction of the thoracolumbar spine when the leak site cannot be identified. After this procedure, bed rest for eight hours in the Trendelenburg position is recommended to allow blood to ascend to the CSF leak [16]. If the patient remains symptomatic, a second EBP should be considered after five days; the EBP is effective in relieving symptoms in one-third of patients [17]. The mechanism of action is unproven. It likely causes a rise in intracranial pressure due to mass effect, leading to short-term symptom relief regardless of whether the underlying CSF leak has healed. The coagulation of blood at the site of the CSF leak may promote the natural healing of the dural tear.

Targeted patching: Once the leak location is identified, targeted patching can be planned for patients who continue to experience symptoms despite conservative measures and untargeted EBP. For this, blood, fibrin sealant, or both can be used; a targeted blood patch is usually preferred compared to placement at other levels. Beard's study suggests that an untargeted blood patch can extend only 3-5 segments from the injection site, so blood injected into the lumbar spine does reach the cervical levels, but the amount needed to form a stable clot is unclear. Potential risks of this technique include cord compression, cranial nerve palsies, especially with cervical epidural patches, seizures, and chemical meningitis. Additionally, using fibrin glue is linked to aseptic meningitis, arachnoiditis, and allergic reactions [3-17].

Surgery

Neurosurgical repair is considered for patients who have not responded to conservative management and who have undergone at least two EBP procedures, with the CSF leak location identified [16,17]. The choice of surgical technique largely depends on the leak's location and specific characteristics, as well as the underlying etiology if identifiable. Case-control studies from spinal centers specializing in CSF leaks have demonstrated excellent outcomes with surgical treatment, resulting in symptom resolution in most cases.

Evolution

Although spontaneous resolution of SIH has been documented within approximately two weeks, a proportion of patients continues to experience persistent symptoms despite radiographic closure of the CSF leak, with manifestations lasting for months or even years [7]. In cases characterized by intermittent leakage at intervals of weeks to months, patients may present with episodic headaches in the absence of additional symptoms between episodes. Furthermore, nearly 10% of patients with spontaneous CSF leaks develop recurrence despite adequate therapeutic intervention [10]. In the present case, the patient demonstrated clinical improvement three months after hospitalization, with complete resolution of headaches and no recurrence of the previously reported symptoms.

While SIH is well-documented, a dural breach secondary to cervical degenerative changes, specifically, osteophytic spurs or herniated discs, remains an outlier in clinical practice. Table 1 provides a comprehensive synthesis of existing literature regarding SIH resulting from cervical osteophytic pathology, detailing the diverse clinical manifestations, diagnostic interventions, and therapeutic outcomes observed in these rare cases.

**Table 1:** The five reported cases of SIH resulting from cervical disc-osteophytic pathology M: male; F: female; ACD: anterior cervical discectomy; EBP: epidural blood patch; ACDF: anterior cervical discectomy and fusion; CSF: cerebrospinal fluid; CT: computed tomography; SIH: spontaneous intracranial hypotension

Authors	Type of report	Years	Age and sex	Clinical presentation	Imaging	Management	Outcomes
Vishteh et al. [7]	Case report	1998	32, M	Postural headaches	Brain MRI: pachymeningeal enhancement and hindbrain herniation. Myelography: extrathecal contrast material ventral to the cervical spinal cord, as well as an unusual midline bone spur at the C5-C6 level	C5-C6 ACD and osteophytectomy, accompanied by meticulous suture closure of the dura using two interrupted Prolene sutures	Asymptomatic, with complete resolution observed in imaging of the meningeal enhancement and mild amelioration of the cerebellar herniation
Eross et al. [8]	Case report	2002	39, F	6-year history of postural headaches, neck stiffness, nausea, and vomiting	Brain MRI: abnormal dural enhancement at the level of the foramen magnum and upper cervical spine. Spinal CT scan: disc herniation and posterior osteophyte at the C7-T1 level	C7-T1 ACDF with primary suture repair of a 3 mm dural defect and placement of an adjunct lumbar drain	Slight alleviation in the severity of headaches. No follow-up imaging findings have been recorded
			44, F	Postural headaches, nausea, ear fullness, and nasal tightness. Paresthesia involving hands and feet	Brain and spinal MRI demonstrated abnormal dural enhancement at the foramen magnum and upper cervical spine, with minimal enhancement over the convexities. CT myelography revealed disc herniation and a posterior osteophyte at C5-C6, with an anterior extra-arachnoid fluid collection spanning C6-T11	Failure of conservative treatment and EBP. C5-C6 ACD and osteophytectomy, then C6 and C7 corpectomy, and lumboperitoneal shunt	The patient remained disabled with orthostatic headaches. No meningeal enhancement, with a persistent collection extending from T2 to T4. CT myelography demonstrated a CSF leak in the anterior epidural space from T3 to T9
			46, M	Several months of postural headaches, neck pain, tinnitus, and recurrent epistaxis	Brain MRI: no evidence of dural enhancement, bowing of the optic chiasm, Chiari malformation, or other signs of brain sagging. Spinal CT scan: CSF leak extending from C6 to T2, a left-sided disc herniation at C4-C5, and a prominent posterior bone spur and superimposed disc just to the right of midline at the C7-T1	Lumbar autologous EBP and propoxyphene	Clinically improved but required propoxyphene. Reduced dural enhancement and epidural venous plexus engorgement, with residual fluid at C2-C5 and C7
Witiw et al. [9]	Case report	2011	46, F	Progressive orthostatic headaches for three months	Brain MRI: bilateral subdural hematomas and pachymeningeal gadolinium enhancement. CT myelogram: anterior epidural CSF collection commencing at the site of a C4-C5 calcified intervertebral disc protrusion. Cervical spinal CT scan: calcified intervertebral disc protrusion	C4-C5 ACD and osteophytectomy. No primary dural suturing was performed; instead, a collagen-based dural substitute membrane was applied and secured with fibrin glue	Asymptomatic, with full resolution of the imaging findings
Our case	Case report	2026	41, M	Two months of recurrent headaches, dizziness, and an acute confusional state	Brain MRI: diffuse meningeal enhancement with brainstem sagging and bilateral subdural hematomas. Spinal CT scan: rostrally oriented osteophyte at the C7-T1 level. Spinal MRI: fluid collection primarily in the anterior upper cervicothoracic region. CT myelography: fluid collection at the cervicothoracic junction with progressive contrast leakage	Surgical evacuation of the bilateral subdural hematomas. C7-T1 ACDF and osteophytectomy with suture closure of the dura	Asymptomatic, with full resolution of the imaging findings

## Conclusions

This case underscores that SIH remains a diagnostic chameleon, particularly when presenting in young, otherwise healthy patients without a history of trauma. The clinical transition from nonspecific flu-like symptoms to subacute neurological deterioration highlights the risk of diagnostic overshadowing, where secondary findings, such as subdural hematomas, may be mistakenly viewed as the primary pathology rather than a consequence of low CSF pressure. Clinicians must maintain a high index of suspicion for SIH in the differential diagnosis of persistent or recurrent headaches, regardless of whether the classic orthostatic component is immediately apparent. Systematic utilization of contrast-enhanced cranial and spinal MRI, followed by targeted myelography, is essential for identifying subtle pachymeningeal enhancement and localizing mechanical causes such as osteophytic bone spurs. Ultimately, this report serves as a reminder that degenerative spinal disease can manifest as a source of CSF leakage even in younger populations, necessitating a comprehensive imaging approach to ensure timely intervention and prevent further neurological decline.
